# The emergence of phonological dispersion through interaction: an exploratory secondary analysis of a communicative game

**DOI:** 10.3389/fpsyg.2023.1130837

**Published:** 2023-05-24

**Authors:** Gareth Roberts, Robin Clark

**Affiliations:** Department of Linguistics, University of Pennsylvania, Philadelphia, PA, United States

**Keywords:** cultural evolution, phonology, combinatoriality, emergence of structure, language, communication, experiment

## Abstract

**Introduction:**

Why is it that phonologies exhibit greater dispersion than we might expect by chance? In earlier work we investigated this using a non-linguistic communication game in which pairs of participants sent each other series of colors to communicate a set of animal silhouettes. They found that above-chance levels of dispersion, similar to that seen in vowel systems, emerged as a result of the production and perception demands acting on the participants. However, they did not investigate the process by which this dispersion came about.

**Method:**

To investigate this we conducted a secondary statistical analysis of the data, looking in particular at how participants approached the communication task, how dispersion emerged, and what convergence looked like.

**Results:**

We found that dispersion was not planned from the start but emerged as a large-scale consequence of smaller-scale choices and adjustments. In particular, participants learned to reproduce colors more reliably over time, paid attention to signaling success, and shifted towards more extreme areas of the space over time.

**Conclusion:**

This study sheds light on the role of interactive processes in mediating between human minds and the emergence or larger-scale structure, as well as the distribution of features across the world's languages.

## 1. Introduction

This paper is concerned with how phonological organization comes about. The phonological inventories of natural languages seem to exhibit structure. Vowel systems are a relatively well-known example of this: If the vowel phonemes of a language are plotted according to their formant values, they tend to exhibit more dispersion and symmetry than might be expected by chance (Liljencrants and Lindblom, 1972; Schwartz et al., 1997; de Boer, 2000). But why should this be?

Certain classes of account explain such organization in terms of *markedness* and *distinctive features* (Jakobson and Halle, 1956; Chomsky and Halle, 1968). These accounts can be understood as framing organization in terms of descriptive simplicity (though see de Boer, 2001; Blevins, 2004 on the danger of circularity in such approaches), while other accounts have attempted to ground distinctive features and markedness in terms of the physical realities of the articulatory system and their constraining influence on individual phonemes (e.g., Flemming, 2001; Stevens and Keyser, 2010; Carré et al., 2017). Other accounts have focused on the functional advantages of dispersion for the system as a whole (e.g., Lindblom, 2003). This account (while not mutually exclusive with the other accounts) emphasizes the role of interactive production–perception dynamics in the emergence of phonological organization, abstracting away from the particular details of the production system in question.

To investigate the role of such processes, Roberts and Clark (2020) employed a non-linguistic communication-game experiment. This kind of approach, termed *Experimental Semiotics* by Galantucci (2009), has become increasingly widely used over the last two decades. It typically involves participants playing games in which they collaboratively construct a novel communication system in the laboratory (e.g., Galantucci, 2005; Fay et al., 2010; Stevens and Roberts, 2019), although the term is also used to include experiments in which participants are given a pre-designed artificial language to learn (e.g., Kirby et al., 2008; Sneller and Roberts, 2018; Wade and Roberts, 2020). The approach was devised primarily to investigate the emergence of language and of linguistic structure and can be distinguished from classic artificial-language learning approaches (e.g., Hudson Kam and Newport, 2009; Culbertson et al., 2012; Fedzechkina et al., 2017) in the inclusion of a social component whereby participants are exposed to each other's communicative output, either directly through interaction (e.g., Galantucci, 2005; Sneller and Roberts, 2018), or—in iterated learning experiments—indirectly through exposure in training to the output of previous participants (e.g., Kirby et al., 2008; Roberts and Fedzechkina, 2018). A principal advantage of the approach is that it allows researchers to incorporate social factors—including genuine interaction—rather directly into experiments while also maintaining a high degree of control (Galantucci and Roberts, 2012; Roberts, 2017). Sender–receiver games in particular are well-positioned to investigate the consequences of pressures acting on interaction—Wade and Roberts (2020), for instance, investigated the role of expectation and observation in driving interactive accommodation in dialog. For our purposes it was also a particularly approach because the task was communicative, but non-linguistic, in nature. This allowed it to shine a light on the role of general, non-language-specific, communicative factors in phonological organization.

**Figure 1:**
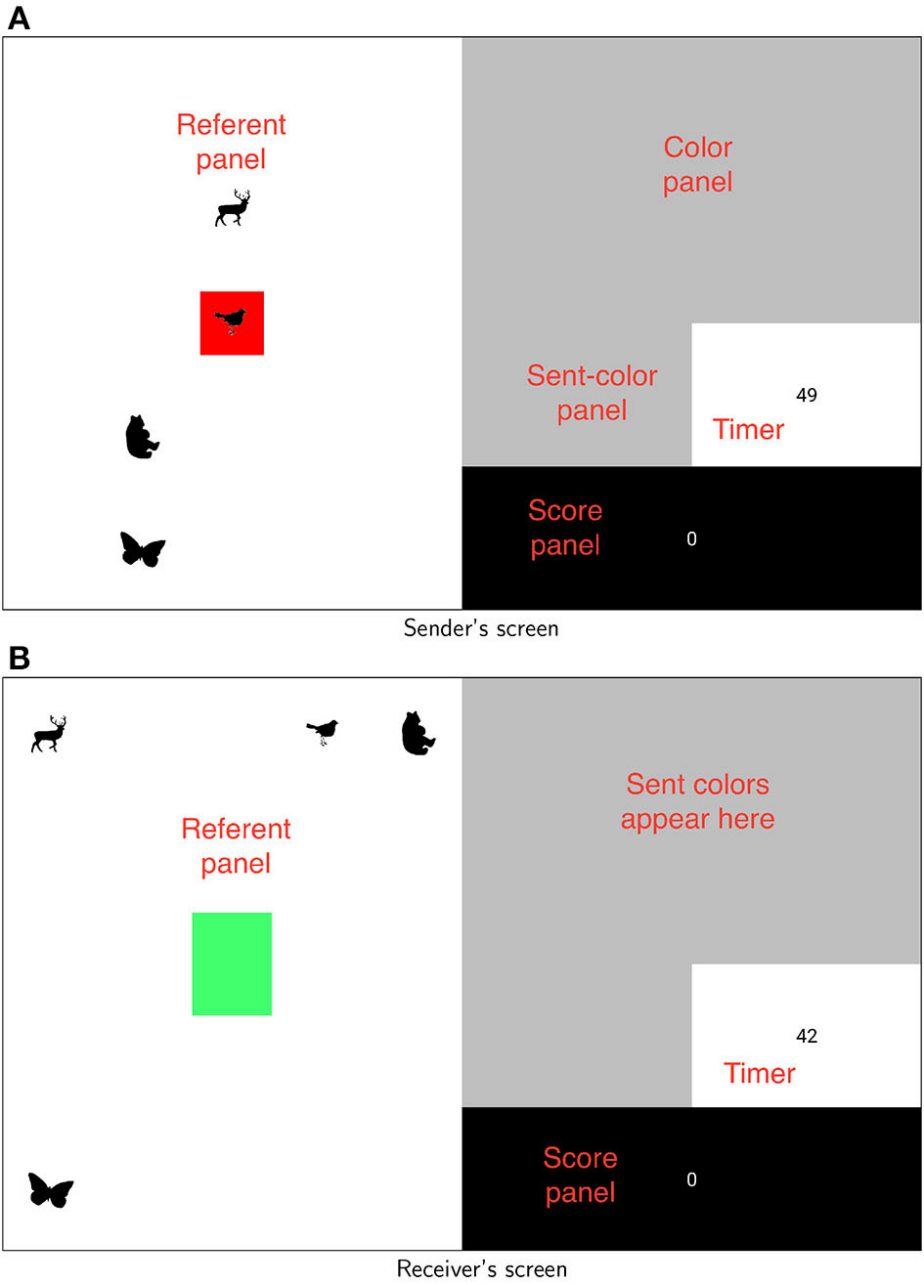
Participants' screens. Labels are for clarity and were not shown to participants. Referents never appeared in the same places on both screens (and no referent ever appeared in the middle space on the receiver's screen). **(A)** Sender's screen. **(B)** Receiver's screen.

In Roberts and Clark's (2020) experiment, pairs of participants took turns to move their fingers around on trackpads to select colors from a continuous colorspace to send to each other, with the goal of communicating a set of *referents* (specifically silhouettes of animals; see Figure 1 for examples). As stated, the non-linguistic nature of the game was crucial; the idea was to observe whether vowel-like dispersion would arise in a novel medium, as this would provide support for non-language-specific accounts. Roberts and Clark (2020) also manipulated the extent to which the production demands acting on the sender and the perceptual demands of the receiver were aligned as a means of identifying the role of these demands in the emergence of structure.

In this paper we report new exploratory *post-hoc* analysis of the data from this experiment. Roberts and Clark (2020) presented results on such dependent variables as participants' success at the game as well as the level of dispersion in their communication systems. However, they did not discuss how the communication systems developed, how participants approached the (non-trivial) communication task they were faced with, how dispersion arose, or how participants converged with each other. Here we examine these questions, which we consider to be interesting and important for a fuller understanding of how structure comes about. Did participants, for instance, privilege dispersion from the beginning of the game, or did it emerge over time as a self-organizing feature, as a result of smaller-scale goals (cf. Lindblom et al., 1983; Keller, 2005)?

Section 2 will first lay out the basic details of the original experiments. The following sections will then discuss the new exploratory analysis. In general this analysis will focus on patterns across all pairs of participants and attempt to shed light on how the participants initially approached the game, how pairs converged with each other, and how organization (principally dispersion) arose.

## 2. Description of experiment

### 2.1. Overview of method

**Figure 2:**
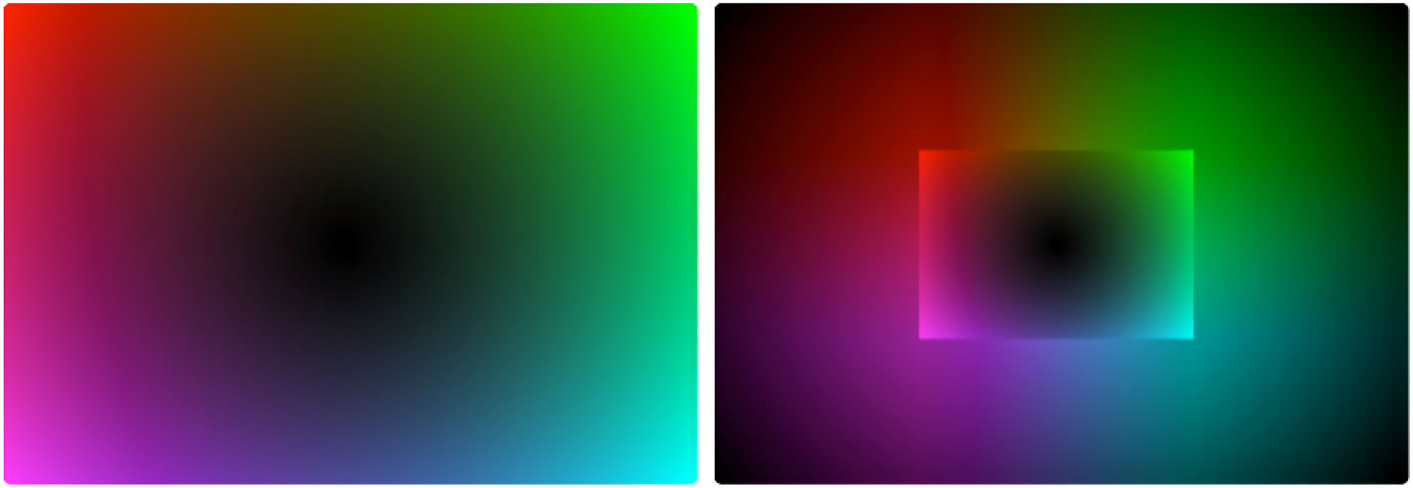
Example color spaces for outer-edge and inner-edge conditions, respectively. Two points should be noted. First, participants never saw the space itself, only individual colors. Second, it is an artifact of this representation that colors drawn from the center area of both spaces appear more indistinguishably dark than they in fact were.

A detailed account of the method is provided in Appendix 1. The basic idea is that pairs of participants played a cooperative referential communication game on computers. The game involved taking turns as *Sender* and *Receiver* in communicating a set of animal silhouettes (Figure 1). At the start of the game, four animal referents were visible on the left of the screen (later, more would be added). Every turn one of these animals would be marked for the sender as the referent that needed to be communicated that turn. Players could not see or hear each other and so the sender had to communicate via a non-linguistic medium. In particular, they could communicate by moving their finger around on a trackpad. Finger positions (which were recorded as *xy* coordinates) corresponded reliably to points on an underlying color space (Figure 2). Participants never saw the whole underlying colorspace; however, as the sender moved their finger around, different colors (which were recorded as RGB values) would appear on their screen. If they held their finger in place for 1 s the color would be sent to the receiver and would appear on their screen. (see Figure 15B in Appendix A for an example.) The sender could select and send as many colors as they wished within the available time of 20 s per round. Before the round was up the receiver could use arrow keys to select the referent they thought the sender was trying to communicate. Feedback was provided to both players at the end of the round. As pairs got better at communicating referents (specifically when every current referent had been communicated successfully on at least three of the previous four rounds where it had occurred) four new referents would be added up to a total of 12. (The full set of referents can be seen in Figure 15A in Appendix A)

Because we were interested in the role of a trade-off between the sender's ease in reliably and consistently selecting colors to send and the receiver's ease in distinguishing colors sent to them, we manipulated how well these pressures lined up. In the *Outer-edge condition* colors became more brighter and more distinct the further the sender's finger was from the center of the pad. This meant that the clearest colors for the receiver were also the easiest to locate consistently. In the *Inner-edge condition* colors initially became brighter and more distinct before abruptly getting darker and less distinct again. This meant that the best colors for the receiver were harder to locate consistently (Figure 2). The most convenient parts of the pad for the Sender to select reliably were still along the outer edge of the pad, but the easiest colors to distinguish for the Receiver were closer to the inner edge. The inner edge was in no way marked on the pad or screen; it became apparent to the Sender as they moved their finger around the pad and observed the effect.

### 2.2. Summary of original analysis and results

Participants' behavior in the communication game created sets of *signs*. By sign we mean a pairing of a referent (i.e., one of the animal silhouettes) with a signal (a series of colors). Each signal consisted of two sets of coordinates, a set of *xy* coordinates corresponding to the sender's finger position on the trackpad and a set of RGB coordinates corresponding to the color that appeared on screen. Because the RGB coordinates for any given trial can be straightforwardly derived from the *xy* coordinates, and the patterns of results for the two spaces are thus the same for many dependent variables, Roberts and Clark's (2020) analysis focused primarily on the *xy* coordinates, which—being two- rather than three-dimensional—are simpler to deal with. We will do the same in this paper. The main exception concerns the *mode brightness* measure, described below. This will be presented separately.

Roberts and Clark (2020) identified inventories for each pair of players by pooling the colors used by each participant (across signals) and calculating Pillai scores to identify “color phonemes” (Hay et al., 2006; Hall-Lew, 2010; Nycz and Hall-Lew, 2013).^1^ They then looked at a series of measures, including—most importantly—*dispersion* and *success*. Dispersion was measured in three different ways: mean pairwise distance (in terms of *xy* coordinates) between phonemes in an inventory; mean distance of *xy* coordinates from the center of the space; and mode brightness. Mode brightness meant the mean value of the brightest RGB component in each phoneme and was a perceptual analog of the distance-from-center measure.^2^ These measures could then be compared with chance-level values, which were calculated by randomly generating 100,000 inventories (for which the mean value is indicated on **Figure 4** by a red dotted line; see Roberts and Clark, 2020, p. 132–133, for more details.)

**Figure 3:**
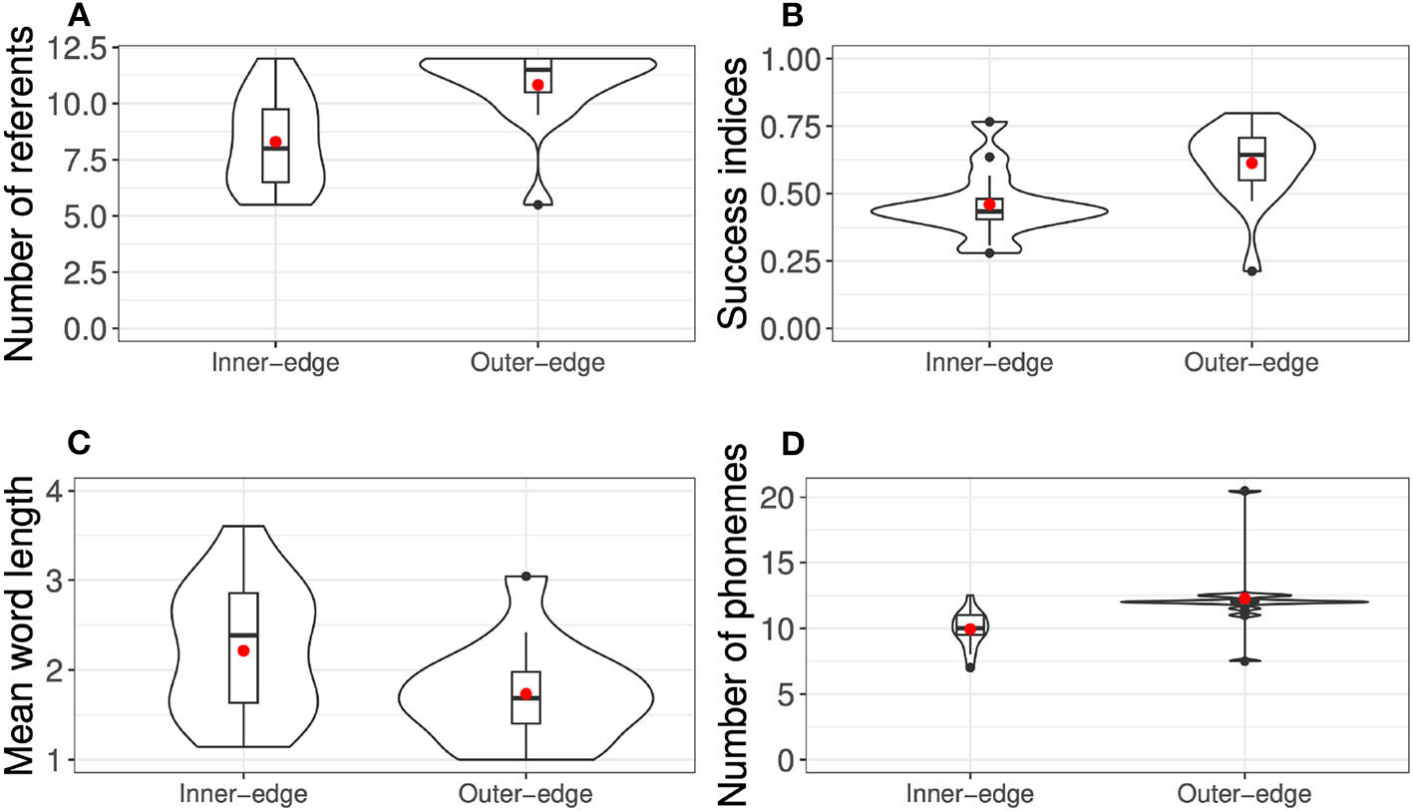
Violin plots of non-dispersion results from original experiment, overlaid with bar and whisker plots. **(A)** Number of referents. **(B)** Success indices. **(C)** Mean word length. **(D)** Number of phonemes. Red dots indicate means.

**Figure 4:**
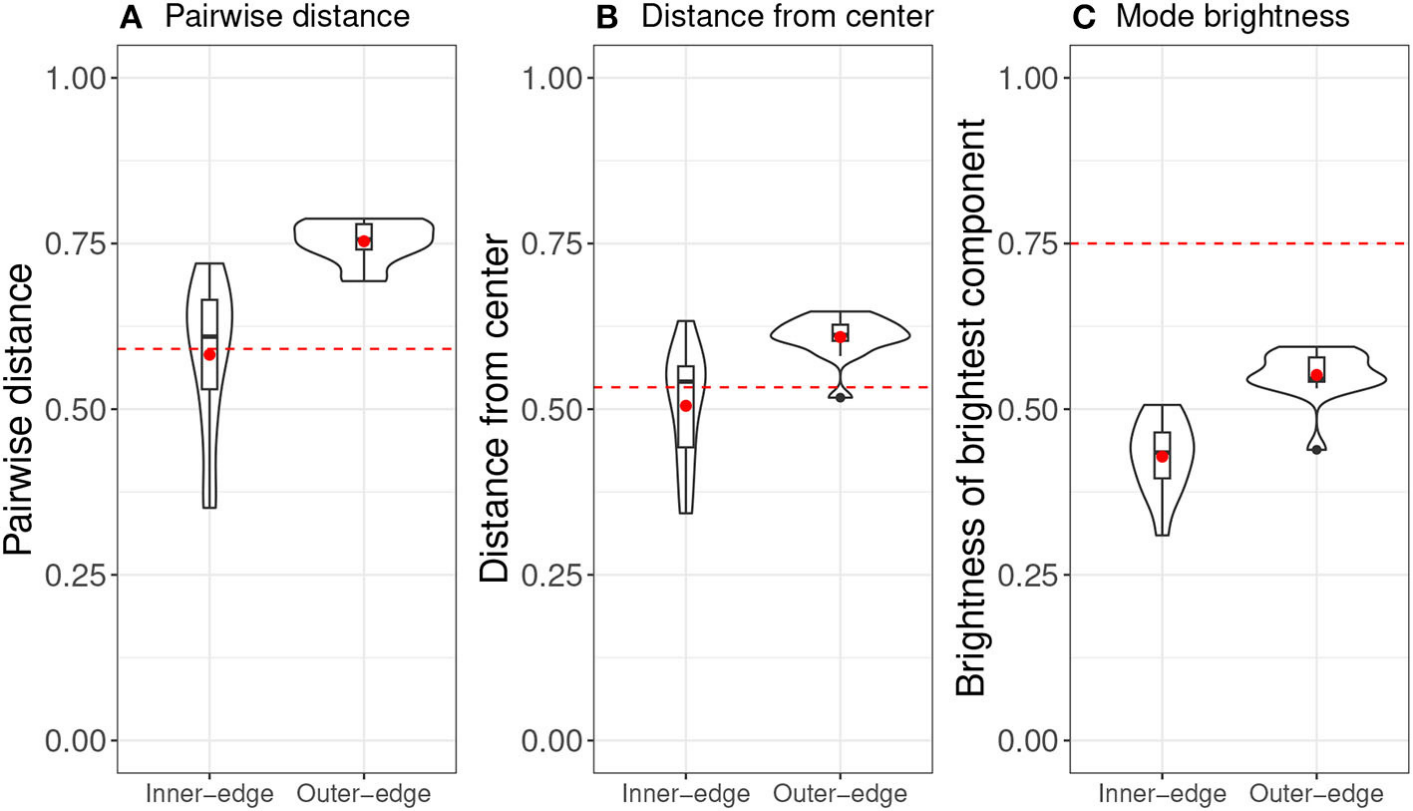
Violin plots of dispersion results from original experiment (Roberts and Clark, 2020), overlaid with bar and whisker plots. Red dots indicates means and red dotted lines indicate chance level. **(A)** Pairwise distance. **(B)** Distance from center. **(C)** Mode brightness.

Success was measured by first counting, for every round of a given game, how many referents each player had established a signal for at that point (establishing a signal meant communicating it successfully in at least three of the last four rounds in which it had occurred). The success index was then calculated as $\left(\overset{n_{r}}{\underset{i=1}{\sum }}s\right)/12n_{r}$, where *n*_*r*_ is the number of rounds and the numerator is a cumulative count of *s*, the number of successfully established words in a given round, with 12 being the maximum possible given the number of referents.^3^ We also measured the number of established signals at the end of the game, the mean word length, and the number of phonemes in players' inventories. The results of all these measures are presented in Figures 3, 4.

**Figure 5:**
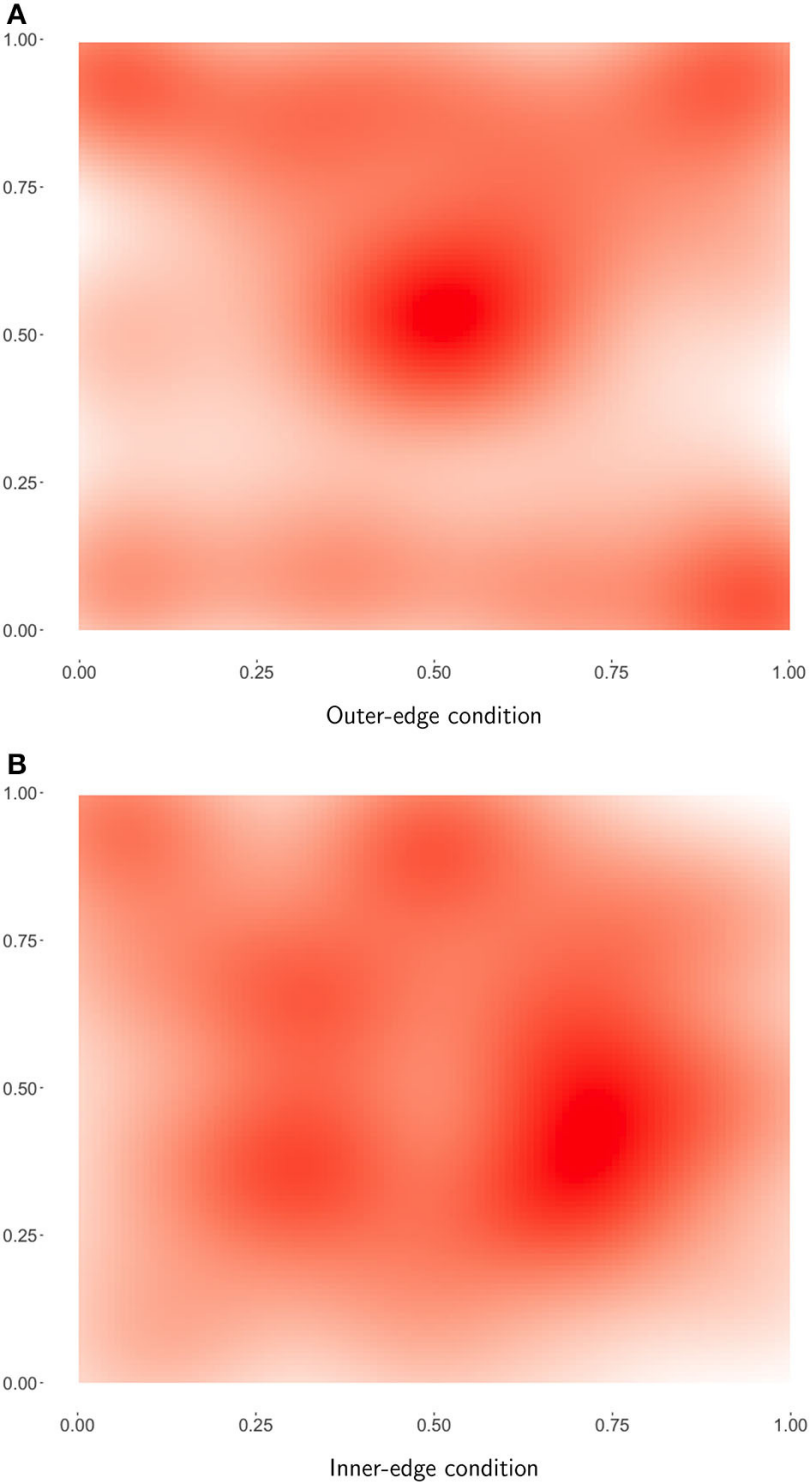
Heat maps of final successful referents. Darker red indicates that this area of the space was more commonly used. **(A)** Outer-edge condition. **(B)** Inner-edge condition.

Overall the results indicated that dispersion qualitatively analogous to that seen in natural-language vowel systems had indeed emerged. This can be seen particularly well in Figure 5, which shows heat maps of final phoneme sets across pairs. A comparison of the two conditions suggested that the pattern of dispersion was driven primarily by perceptibility demands rather than by ease of production. As a result, participants found the Inner-edge condition, in which perceptual demands were misaligned with production demands, significantly more difficult. Success was related to dispersion, but this relationship was only apparent when both conditions were considered together, suggesting that the difference between conditions was driving this relationship.

But how did the patterns observed come about? This was not addressed by Roberts and Clark (2020) and will be discussed in the following sections of this paper.

## 3. New exploratory analysis

As discussed above, each signal that participants produced in the game could consist of several colors. Roberts and Clark (2020) conducted an analysis that compared the various different colors used and generated a phoneme inventory. In principle a dyad might combine quite a small set of phonemes to create a number of distinct signals. For example, four different color phonemes (e.g., one in each corner of the space) could be recombined into enough two-unit signals for all 12 referents in the game. However, this was not in fact a typical approach. Rather, pairs tended to come up with systems with roughly the same number of phonemes as referents they were communicating (Figures 3A, D). There are a few likely reasons why this is the case. First, producing more than one color per referent requires extra effort, so we should expect participants to stick to one if they can. Second, as can be inferred from the fact that pairs employing this strategy were able to do well, the communication medium afforded enough distinct colors to communicate all referents available. Third, this effect was likely bolstered by the fact that participants initially had only four referents to communicate—this put even less pressure on them to combine colors, and so they were unlikely to be in the habit of doing so when more referents were added. To an extent then, this result was an artifact of the task design. However, such effects are not unprecedented in natural language: ABSL is a well-known example of a language that apparently lacked combinatorial phonology—by which is meant meaningless units reused between signs—for a surprisingly long time (Sandler et al., 2011). It has long been argued that phonology likely emerges as the set of signs increases in size, leaving less space for distinct signs in the absence of recombination (e.g., Hockett, 1960). However, several experimental studies have failed to find strong evidence that the number of signs plays a very important role, with evidence instead that capacity for iconicity (i.e., the extent to which the medium affords iconic signs) and ease of articulation (i.e., how easy it is to expand the phonological inventory) may play more important roles, at least in early stages (Roberts and Galantucci, 2012; Verhoef et al., 2014; Roberts et al., 2015).

As the relationship between phoneme inventory size and referent set size in our data might suggest, a closer examination of the sign sets in our data revealed that most signals tended to consist of one color repeated several times rather than combinations of more than one color. For this reason our analysis in this paper will dispense with Roberts and Clark's (2020) phoneme sets and simply focus on the first color of each signal only. This clearly simplifies our analysis by eliminating the need for any attempt to distinguish distinct but similar phonemes from imperfect repetitions (which is especially difficult for signs for which there are a low number of exemplars); it also expands the number of signals that we can examine over time (as we do not need to abstract over series of signals for the same referent over time, as required by the Pillai score analysis). Furthermore, we consider that an analysis of the distribution of signal-initial colors would itself be illuminating even if were not the case that signals tended to involve repetition.

In what follows we will look at participants' initial behavior as they began playing the game (Section 3.1), how dispersion emerged over time (Section 3.2), and at convergence between partners (Section 3.3). We performed the analyses using R (R Core Team, 2014), and conducted linear mixed effects models using the lmerTest library, which employs the Satterthwaite approximation to obtain a *p*-value from a t-value (Kuznetsova et al., 2017). Where possible (and appropriate given the question being answered), we attempted to include pair and referent as random intercepts and to include random slopes by pair and referent for variables under discussion. In most cases the fully maximal model failed to converge, or reported a singular fit. In such cases we removed random slopes one by one until the model converged. Where there was a choice between which slope to include, we chose based on theoretical importance. The resulting model structure is reported in each case.

### 3.1. Initial behavior

Our first question concerns participants' first signals. How did senders initially approach the task of selecting a signal in an unfamiliar medium? There are several possibilities for how a participant *might* approach it. One would be to privilege audience design. That is, a sender might attempt to take into account the needs of the receiver and select a relatively distinct color, perhaps one that has some iconic relationship with the referent (e.g., brown for a bear), or which is simply a very salient “basic” color (such as bright red). A second possibility is that senders might be driven more by what is easier for themselves, whether by selecting colors at points that are especially comfortable to reach on the trackpad or by selecting colors that will be easy to find reliably in future rounds. The corners of the pad fulfill this last criterion particularly well and also lead to systems that are relatively well-dispersed. Given that the systems participants ended up with in the Outer-edge condition tended to exhibit greater dispersion than would be expected by chance, it could be that they in fact began the game by concentrating on the corners and the center of the trackpad. A third possibility is simply to select randomly. In the first round participants were not yet familiar with the medium and its affordances, so it was not trivial to make decisions that really took into account the needs of either sender or receiver. Selecting a signal randomly is also a good way to start learning about the medium and a reasonable way to start establishing an arbitrary communication system.

**Figure 6:**
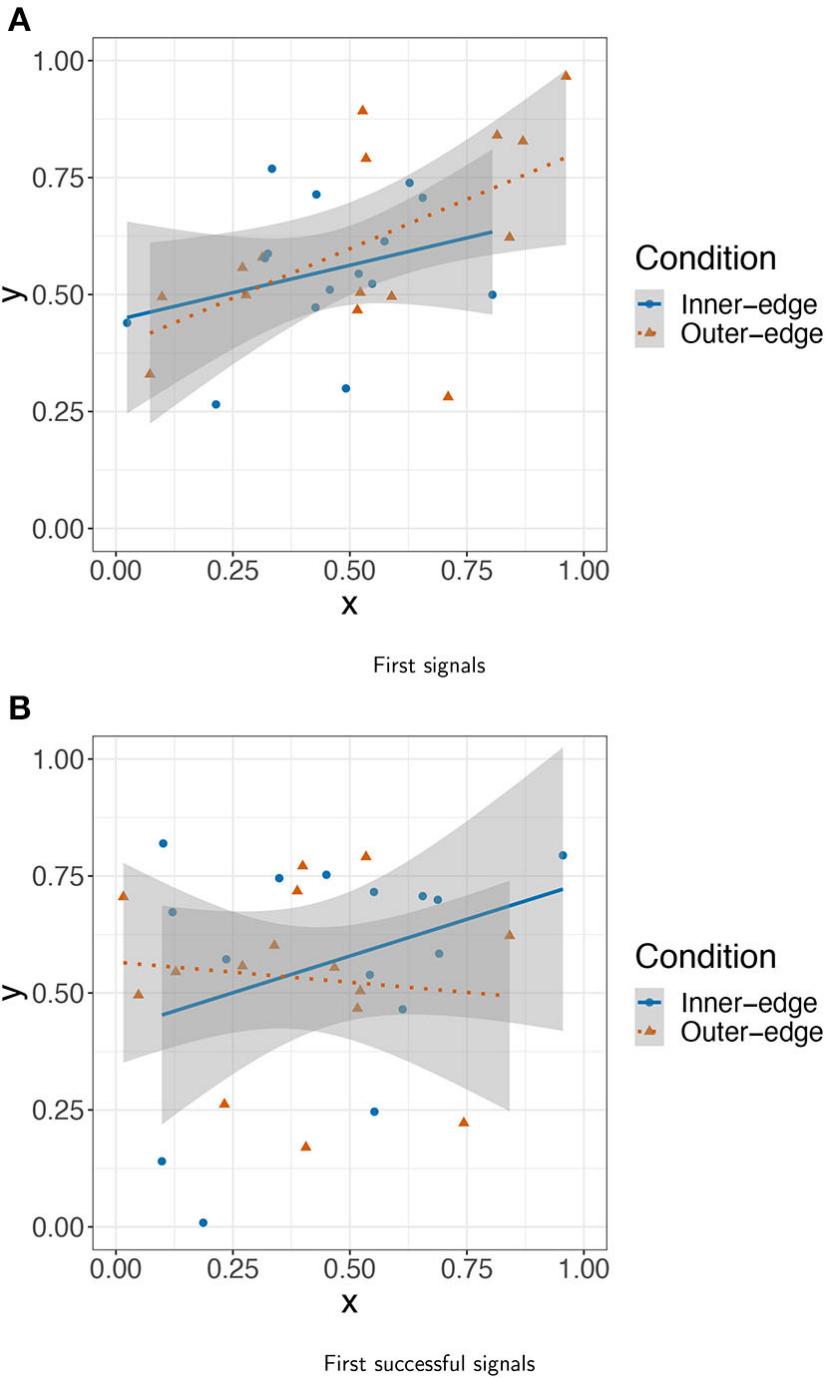
Trackpad location of **(A)** first signal and **(B)** first successful signal for each pair (both conditions).

To investigate what participants actually did, we took the first signal that was sent by every player across both conditions and plotted these signals according to their *x* and *y* coordinates. This is shown in Figure 6A. As can be seen, participants do not seem to have been starting with locations that were likely to help maximize later dispersion (e.g., the corners and center of the pad). In fact the most obvious pattern is that the *x* and *y* coordinates seem positively correlated. To confirm this we performed a mixed model predicting the *y* from the *x* coordinate, with random intercepts for referents, and indeed found evidence of a relationship: β = 0.37, *SE* = 0.12, *t*(26.58) = 3.01, *p* = 0.006. As can be seen in Figure 6, the relationship was stronger for the Outer-edge condition, for which the observed pattern also held true when taken alone, β = 0.397, *SE* = 0.16, *t*(11.87) = 2.45, *p* = 0.03. However, a model of all the data including condition as an interaction term found neither an interaction nor an effect of condition (*ps*>0.1). Overall, while participants were not selecting uniformly random points on the pad, it seems that they might have been selecting random points within an area of the pad stretching from the bottom left side (though not as far as the bottom left corner) to the top right corner. It is tempting to connect this with known human biases to interpret data in terms of positive linear relationships (cf. Kalish et al., 2007). However, what almost certainly matters more here is that this area of the pad is the most physically comfortable area for a right-handed person who is resting the bottom of their palm near the bottom right of the pad. Given this arrangement, the central area of the pad is rather easy to reach. This extends to the top of the pad, but not the bottom. In fact, the whole of the bottom quarter of the pad is hard to reach comfortably with the index finger without moving one's palm. Within the top three quarters of the pad, there is also an asymmetry between the leftmost and rightmost quarters. First, the top-right corner is easier to reach (assuming, as above, a right handed person resting their palm at the bottom right of the pad) than the top-left corner. Below that, however, the index finger has a slightly larger area available to it on the left than on the right. This is because reaching the leftmost area of the pad just below the central horizontal axis merely involves extending one's finger. Reaching the same area on the right (assuming the physical arrangement described above) involves moving one's palm or bending one's finger under the top of the palm. This likely accounts for the space participants drew their first signals from. As for how they selected signals within this space: The particular points selected within this space look rather random. Signals selected in the Inner-edge condition appeared to have a lower mean distance from the center of the pad than those in the Outer-edge condition (0.21 vs. 0.31) but there was no significant difference, t(25) = −1.71, *p* = 0.099. In other words, participants seem to have been driven primarily by physical ease.

This pattern seems to be a feature of initial exploration in particular. We conducted a linear mixed effects analysis as before on (instead of only the very first signal for each player) the first signal for all four of the initial set of referents. The relationship held across conditions, though it was weaker: β = 0.21, *SE* = 0.097, *t*(84.63) = 2.18, *p* = 0.032 Furthermore, the effect disappears if condition is included as in interaction term (*ps*>0.4). But there was no effect for *successful* signals (i.e., the first signal in each pair for which the receiver selected the correct referent; Figure 6B): β = −0.01, *SE* = 0.05, *t*(327.49) = −0.35, *p* = 0.73. In other words, the account given above seems to work as an account of basic starting strategy only. As participants started to get more used to the game and to actually establish a communication system, they seem to have explored more of the space (perhaps beginning to more readily move their palms). To investigate whether this was part of a general trend to use more of the space over time, we conducted a model with distance from center as dependent variable, turn number and condition as fixed effects, condition as an interaction term, and random intercepts for pair and referent. There was an effect of both turn number, β = 2.11 × 10^−4^, *SE* = 1.91 × 10^−5^, *t*(1.25 × 10^4^) = 11.03, *p* < 0.001, and of condition, β = −0.102, *SE* = 1.224 × 10^−2^, *t*(40.4) = 11.03, *p* < 0.001, and an interaction with condition: β = −1.127 × 10^−4^, *SE* = 2.361 × 10^−5^, *t*(1.25 × 10^4^) = −4.78, *p* < 0.001. In other words, participants did indeed use more space over time, but more in the Outer-edge condition—where colors got reliably less dark toward the outer edges of the pad—than in the Inner-edge condition.

As can be seen in Figure 6B, however, participants' first successful signals still do not appear to have been established with an eventually well-dispersed system in mind; there is no evidence, for instance, that participants were preferentially establishing signals on the edges or corners of the space.

In summary then, the apparent picture is as follows. Participants seem to have begun by exploring the most accessible area of the pad and selecting relatively distinct colors from within that space. As they became more familiar with the game, they explored a larger area of the pad. But there is little evidence that they implemented any more coordinated plan to maximize overall dispersion in their emerging system. This is consistent, in other words, with accounts of phonological structure as an emergent, self-organizing phenomenon (Lindblom et al., 1983; Wedel, 2003). In terms of Keller's (2005) account of language change we should think of dispersion as a *phenomenon of the third kind*: an epiphenomenal, large-scale consequence of deliberate smaller-scale behaviors, as opposed to being a directly intended consequence of human decisions or a “natural” phenomenon not caused by human actions.

In the next section we discuss in more detail what this looked like.

### 3.2. Emergence of dispersion over time

In general, as can be seen in Figure 4, pairs in the Outer-edge condition tended to end up with more dispersed systems than would be expected by chance. The general pattern can be seen rather clearly in Figure 5A, which shows a heatmap of final successful signals across pairs in this condition. A comparison with the underlying color spaces in Figure 2 indicates that, while perceptual distinctiveness seems to have driven a great deal of participants' behavior, participants were not simply selecting points in the space that afforded particularly *bright* colors. If that were so, the center of the space would not be as favored as it apparently was. Rather, signals seem to be distributed across the space in a way that increases dispersion, with a slight bias for the top over the bottom of the pad. (see Section 3.1 for a discussion of how this bias might arise from the location of participants' hands.) The pattern for the Inner-edge condition, shown in Figure 5B, suggests that—while systems in this condition were not more dispersed than we would expect by chance—this may be an artifact of participants avoiding the corners of the space, which in this condition were dark (Figure 2). The fact that participants in this condition made much less use of the center than participants in the Outer-edge condition is notable and seems likely driven by a bias for maintaining distance between signals.

**Figure 7:**
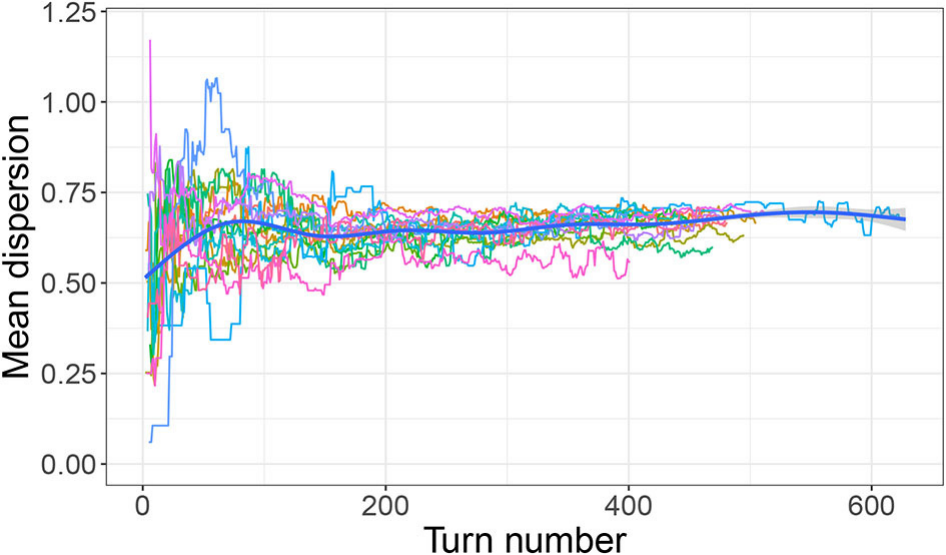
Mean dispersion over time (outer-edge condition). Each colored line indicates dispersion levels (measured as the mean distance between the most recent set of successful signals) for a single pair. Thick blue line indicates smoothed conditional mean.

**Figure 8:**
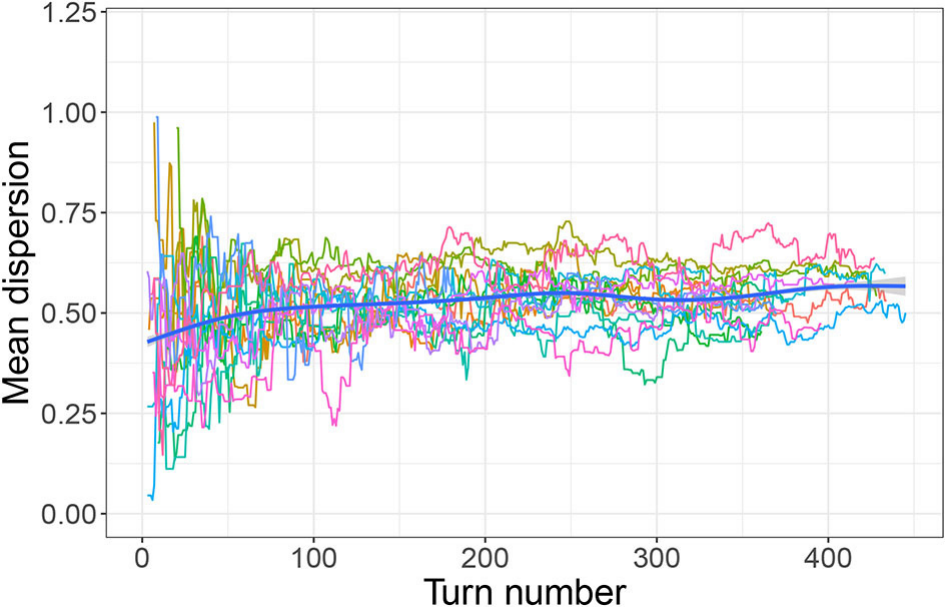
Mean dispersion over time (inner-edge condition). Each colored line indicates dispersion levels (measured as the mean distance between the most recent set of successful signals) for a single pair. Thick blue line indicates smoothed conditional mean.

However, as discussed in Section 3.1, there is little evidence that participants in any condition were directly targeting a high *mean distance* or that they planned from the beginning to create well-dispersed systems. Rather, system-wide dispersion seems to be a feature that emerged over the course of the experiment, most likely as a result of participants simply trying to keep new signals distinct from already established ones. Figures 7, 8 are of interest in this respect. They show mean dispersion (operationalized as the mean distance between all successful signals) over time in the Outer-edge and Inner-edge conditions, respectively. The pattern for most pairs is of an initial increase in dispersion levels over (roughly) the first 75 turns and then a plateau. For some pairs, however, dispersion decreased—in part as a result of having to accommodate new referents. In fact, it is rather interesting that there seems to have been a broadly optimal level of dispersion that pairs converged on. For the Outer-edge condition overall mean dispersion for the whole game was 0.65. Given that the maximum possible distance for two signals (i.e., the distance between coordinates 0,0 and 1,1) is 1.41, this means that the typical situation in the Outer-edge condition was to settle for most of the game on a level of dispersion that was close to half that, which is a rather high level of dispersion for larger sets.^4^ The other notable feature is that levels of dispersion began as very variable, but variability reduced over time. This happens to a great extent in the Outer-edge condition. It also happened in the Inner-edge condition, but to a much smaller degree.

#### 3.2.1. Increasing consistency

**Figure 9:**
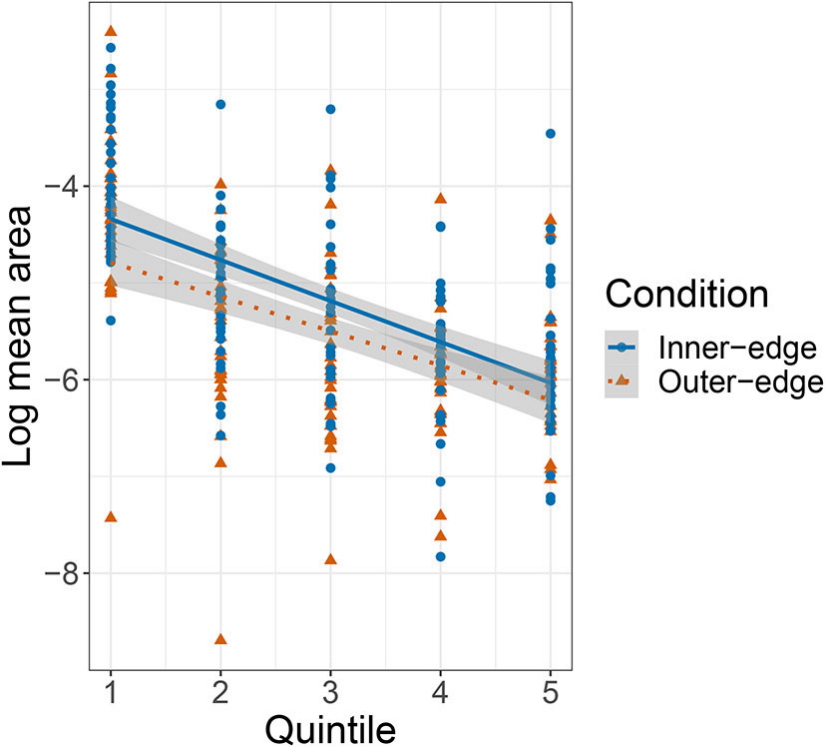
Plot of relationship between game stage (quintile) and the log-transformed mean area covered by signals for each referent. Area is normalized such that the total area of the space was 1^2^ = 1.

How did this reduction in variability come about? In large part it seems likely to have been driven simply by participants becoming more consistent and reliable in selecting signals; that is, by them becoming increasingly likely to hit close to the same point on the trackpad. We investigated this by taking each pair and dividing their series of turns into five equally sized sections (quintiles). For each quintile, the *signal area* for each referent was calculated as follows. First, the coordinates were plotted for all successful signals that had been used to refer to that referent during that quintile. This can be termed the *coordinate cloud* for that pair, referent, and quintile. (Outliers more than two standard deviations from the mean were removed.) To simplify calculating the area of the coordinate clouds, we normalized the slope of each cloud by projecting it onto its first two principal components. The area of the cloud could then be simply calculated as the area of an ellipse whose width was the distance between the lowest and highest valued *x* coordinates and whose height was the distance between the lowest and highest *y* coordinates. Then we calculated the mean area of all coordinate clouds in the quintile.^5^ Figure 9 is a plot of mean areas by quintile. We performed a mixed effects model with mean area as the dependent variable, quintile and condition as fixed effects, condition as an interaction term, and a random intercept for pair. Given the nonlinear nature of the data, we first performed a log-transformation of the mean area. There was a significant effect of quintile, β = −0.42, *SE* = 0.05, *t*(266.4) = −9.06, *p* < 0.001, and of condition, β = −0.45, *SE* = 1.18 × 10^−3^, *t*(104.05) = −2.43, *p* = 0.017, but no interaction between quintile and condition (*p* = 0.29). The pattern is essentially of smaller areas (or, to put it another way, increased precision) from the second quintile onwards. To a great extent this is likely driven by participants' growing familiarity with the game: As they got more practiced at selecting and sending signals, their consistency improved. However, it is also the case that, as they got better at playing the game, they succeeded at communicating more referents, and the number of referents they had to communicate increased. This means that, as participants got more practiced and precise—such that the area of the pad claimed by any given referent decreased—the number of referents with a claim to some space also increased, creating a further pressure to use the pad more economically.

**Figure 10:**
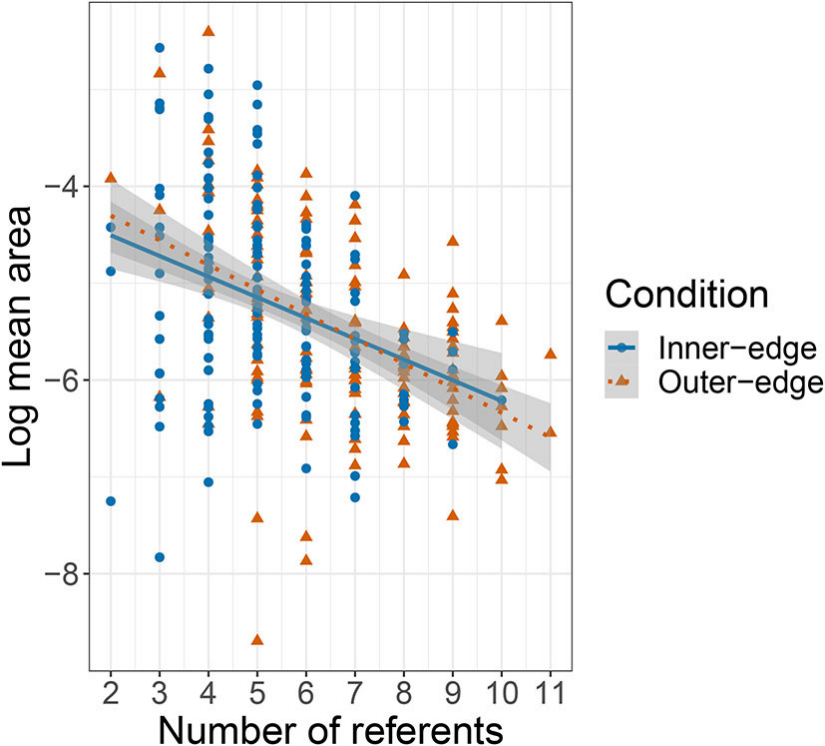
Plot of relationship between number of referents and the log-transformed mean area covered by signals for each referent (based on five quintiles).

Figure 10 shows mean area plotted against the number of referents that participants had successfully communicated. The relationship looks similar to that shown in Figure 9 for mean area by quintile. We performed an equivalent model and found an effect of number of referents, β = −0.21, *SE* = 0.05, *t*(116.9) = −4.53, *p* < 0.001, but no effect of condition, and no interaction (*p*>0.4 in both cases). The apparent pattern is of an initial increase in signal area as participants successfully communicated more referents (and thus had more to keep track of) followed by a decrease as the number of referents they were successfully communicating passed five. Participants did not see a fifth referent until they had successfully communicated each one of the first four referents in at least three out of the preceding four attempts. In other words, participants should have been rather used to the game and doing reasonably well by this point. Successfully communicating six referents meant that they had not only consolidated their grip on the first four referents but had managed to incorporate two more into their system. As a further indicator of increasing reliability, we also measured the distance between each signal and the most recent previous signal for the same referent by the same player (which we will term *auto-distance*). We then conducted a linear mixed effects model with auto-distance as dependent variable, turn number and condition as fixed effects, condition as an interaction term, random intercepts for pair and referent, and a random slope for condition by referent. This revealed a negative effect of turn number, β = −2.79 × 10^−4^, *SE* = 1.89 × 10^−4^, *t*(7.17 × 10^3^) = −14.71, *p* < 0.001, but no effect of, or interaction with, condition (*p*>0.27 in both cases).

Along similar lines, later added referents seemed a little more stable over the course of the game. That is, the mean signal area was slightly smaller for the second set of four referents than for the first and smaller again for the third set (0.026 for the first, 0.019 for the second, and 0.013 for the third). We investigated this further using a linear mixed-effects model with area as the dependent variable and set number, quintile, and condition as fixed effects as well as interactions with condition and random intercepts for pair and referent. This revealed that the effect was driven by quintile (i.e., game stage), β = −2.42 × 10^−3^, *SE* = 3.23 × 10^−4^, *t*(1.74 × 10^3^) = −7.51, *p* < 0.001, rather than by referent set (*p* = 0.15). There was also an effect of condition, β = 4.97 × 10^−3^, *SE* = 1.63 × 10^−3^, *t*(68.6) = 3.06, *p* = 0.003, and an interaction between condition and quintile, β = −1.21 × 10^−3^, *SE* = 4.76 × 10^−4^, *t*(1.74 × 10^3^) = 2.55, *p* = 0.01.

This suggests that earlier introduced signals moved around the space a little more than later established signals, owing primarily to having been introduced earlier. It is perhaps interesting that the earlier established signals did not move more—it does not seem to be the case, for instance, that participants were making *dramatic* alterations to their signal systems to accommodate new signals. This is, however, understandable if one considers the communicative cost of altering an established system. We might, however, expect that some reorganization of this kind—which would increase systematicity—might occur if systems produced by the pairs were taught to new participants, especially in an iterated-learning design (where several generations learn from the output of earlier ones). This has been shown across a number of experiments and simulations to increase systematicity in communication (and non-communication) systems (Kirby et al., 2014; Verhoef et al., 2014). It is also consistent with patterns observed in the emergence of new sign languages outside the laboratory (Senghas et al., 2014), as well as work on chain shifts in the phonologies of well-established languages (Stanford and Kenny, 2013; D'Onofrio et al., 2019).

#### 3.2.2. Extremeness and dispersion

**Figure 11:**
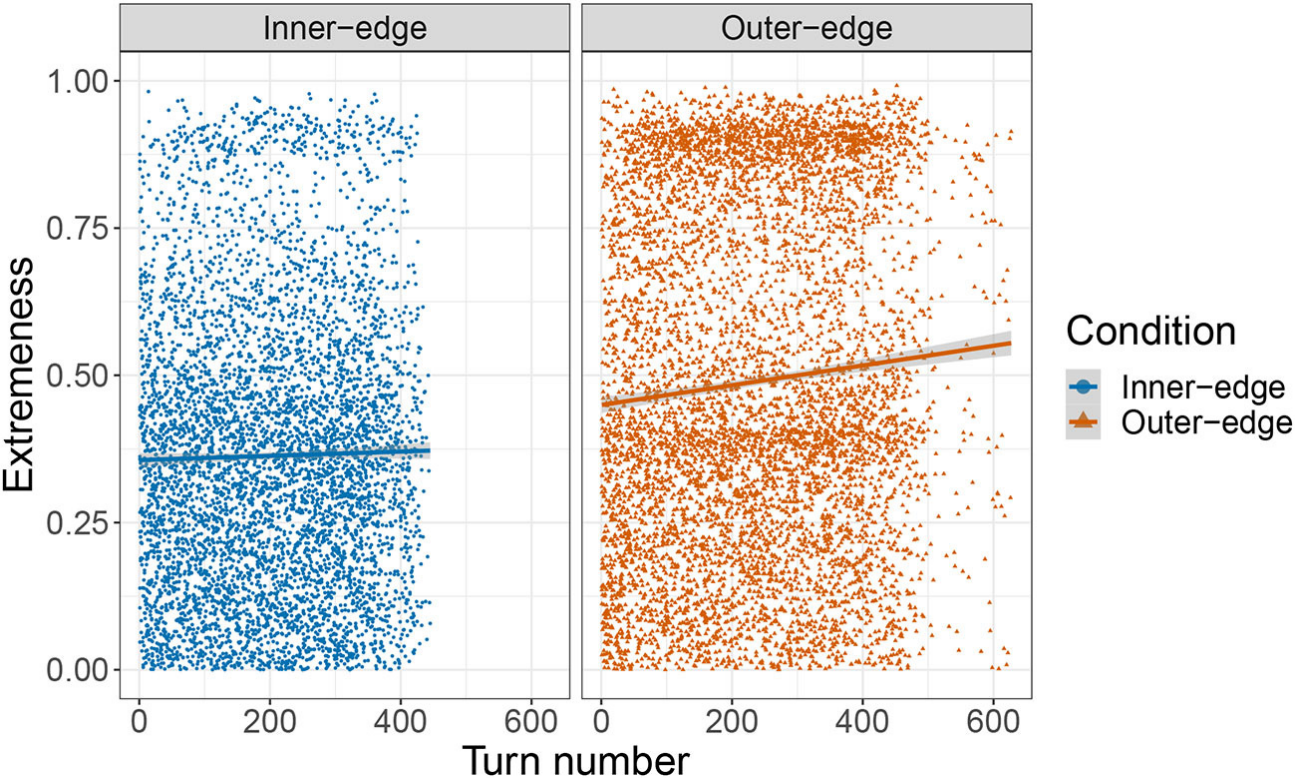
Plot of extremeness index by turn number, faceted by condition.

**Figure 12:**
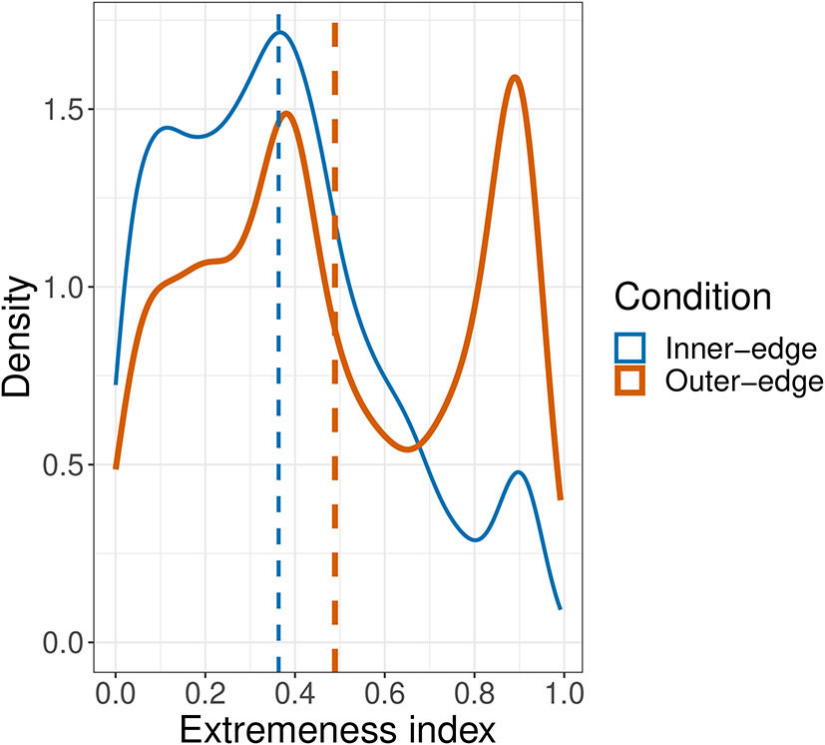
Density plot of extremeness index values. Dashed lines indicate mean values.

So if players did not begin the game by preferentially establishing signals in the corners and center of the space and did not move their initial signals around very much after establishing them, was there a point when they did start preferentially selecting such areas for signals? Was this perhaps more of a late-game phenomenon? We investigated this by calculating an *extremeness index* for every signal. This was simply $\frac{\left|norm.dist-0.5\right|}{0.5},$ where *norm*.*dist* was the distance from the signal to the center of the space normalized by being divided by the maximum distance (i.e., the center to the corner). This resulted in a value between 0 and 1, where a signal in either the absolute center or corner of the space would score 1 and a signal exactly halfway between the corner and the center would score 0. We then looked at whether there was a relationship between the extremeness index and turn number. We conducted a linear mixed effects model with extremeness as dependent variable, turn number and condition as fixed effects, condition as an interaction term, and random intercepts for pair and referent. This revealed a relationship between turn number and extremeness, β = 1.43 × 10^−4^, *SE* = 3.04 × 10^−5^, *t*(1.26 × 10^4^) = 4.72, *p* < 0.001, an effect of condition, β = −0.11, *SE* = 1.88 × 10^−2^, *t*(41.8) = 5.7, *p* < 0.001, and an interaction between turn number and condition, β = −8.12 × 10^−5^, *SE* = 3.75 × 10^−5^, *t*(1.25 × 10^−5^) = 2.17, *p* < 0.001. However, as can be seen in Figure 11, it would be rather misleading to say that there was any very *clear* tendency to select increasingly extreme locations for signals as the game went on. Participants in fact selected extreme locations throughout the game. There was a rather clearer pattern in the overall distribution of extremeness values that can be seen more easily in the density plot in Figure 12. This reveals a bimodal distribution for the Outer-edge condition, with the largest peak at roughly 0.9 (close to the center or corners of the space) and another, only slightly smaller peak, at ~0.4, a value consistent with points on or near the edges—but not corners—of the space. In other words, there was a general tendency throughout the game to select colors in locations around the edge of the pad. There was a peak at 0.4 for the Inner-edge condition too, but only a very small peak at 0.9. Nonetheless, the existence of even a small peak at 0.9 suggests that the advantages to the sender of selecting points in the corners and center of the space played a role even in this condition, where these areas did not correspond to very distinct colors (Figure 2). In this context it is important to emphasize that extremeness and dispersion are related to the number of referents that pairs are trying to communicate. As that goes up, the available space comes to be increasingly occupied. After a certain point (i.e., after the corners, and then the center, have all been taken), mean dispersion and extremeness will inevitably decrease.

### 3.3. Convergence between partners

**Figure 13:**
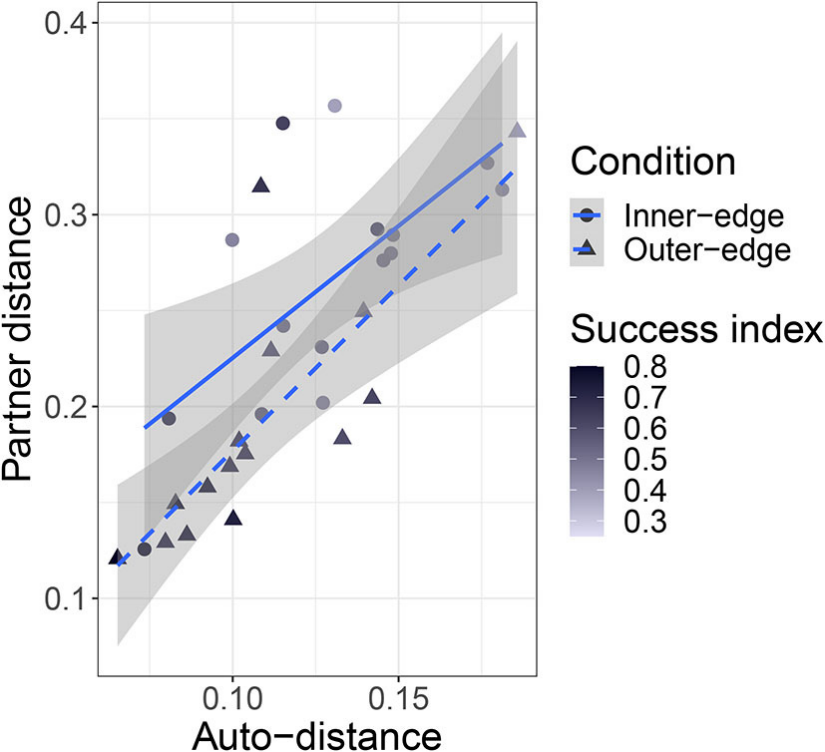
Plot of relationship between partner distance and auto-distance. Shapes and line types indicate condition. Points are colored according to the pair's success index, with darker shades indicating higher success.

In Section 10 above we reported that auto-distance (i.e., the distance between successive signals for the same referent by the same participant) tended to go down over time. The same is true for *partner distance*, by which we mean the distance between a given signal and the last signal for the same referent produced by the other member of the pair. We conducted a linear mixed model with partner distance as the dependent variable, turn number and condition as fixed effects, condition as an interaction term, random intercepts for pair and referent, and a random slope for condition by referent. We found an effect of turn number, β = −2.48 × 10^−4^, *SE* = 2.5 × 10^−5^, *t*(1.12 × 10^4^) = −9.91, *p* < 0.001 and an interaction with condition: β = −1.26 × 10^−4^, *t*(1.16 × 10^4^) = −4.03, *SE* = 3.14 × 10^−5^, *p* < 0.001, suggesting that the relationship between turn number and partner distance was stronger in the Outer-edge condition. More interestingly, mean auto-distance and mean partner distance were very well-correlated across pairs: *r*(28) = 0.75, *p* < 0.001 (Figure 13), suggesting that more consistent participants were also more likely to do a good job of aligning with their partners. There was also a negative relationship between partner distance and success. We performed a linear mixed effects model with pair distance as dependent variable, success index and condition as fixed effects, condition as an interaction term, random intercepts for pair and referent, and a random slope for condition by referent. There was an effect of success, β = −0.43, *SE* = 0.13, *t*(26.05) = −3.23, *p* = 0.003, but no effect of, or interaction with, condition. This supports the intuition that consistency and alignment were beneficial to performance in the game, regardless of condition.

One other thing to consider is that the relationship between pair-distance and auto-distance might itself be of importance. A player who was highly consistent with themselves but who never followed the lead of their partner might drag down success in spite of their low auto-distance. However, a comparison of the ratio between partner distance and auto-distance with success index did not yield evidence of a relationship. This is not too surprising given the close relationship between partner distance and auto-distance discussed above. As can be seen in Figure 13, there are in fact very few points under the regression line (indicating higher than average auto-distance relative to partner distance); nor were they especially unsuccessful. There is also no particularly clear success pattern to be seen among the participants with high partner distance relative to auto-distance.

**Figure 14:**
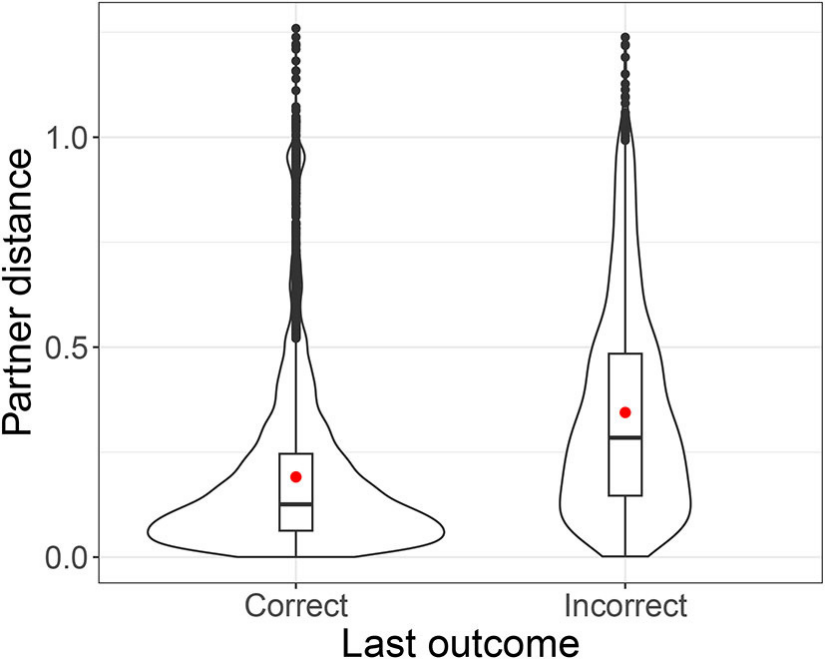
Violin plot of partner distance by success of previous signal for same referent, overlaid with bar and whisker plots. Red dots indicate means.

How did pairs converge? Part of the story is that players paid attention to success. In general partner distance was smaller if the last signal for the same referent was successful (Figure 14). A mixed model with partner distance as dependent variable, last outcome and turn number as fixed effects, their interactions with condition, pair and referent as random intercepts, and random slopes for last outcome by referent, found an effect of the last outcome being correct, β = −6.82 × 10^−2^, *SE* = 2.83 × 10^−2^, *t*(16.6) = −2.41, *p* = 0.028, an effect of turn number, β = −1.66 × 10^−4^, *SE* = 2.37 × 10^−5^, *t*(9.42 × 10^3^) = −6.99, *p* < 0.001, an interaction between last outcome (correct) and condition, β = −0.15, *SE* = 4.09 × 10^−2^, *t*(2.48 × 10^3^) = −3.63, *p* < 0.001, and an interaction between turn number and condition, β = −1.43 × 10^−4^, *SE* = 2.93 × 10^−5^, *t*(1.18 × 10^4^) = −4.88, *p* < 0.001. For auto-distance, we also found an effect of last outcome (correct), β = −0.11, *SE* = 1.57 × 10^−2^, *t*(1.19 × 10^4^) = −6.73, *p* < 0.001, and of turn number, β = −2.24 × 10^−4^, *SE* = 1.77 × 10^−5^, *t*(1.17 × 10^4^) = −12.62, *p* < 0.001, but no effect of condition and no interactions. In other words, when pairs had signaled successfully, they generally tried to stay close to what had worked; when they were unsuccessful they tried something new.

As might be expected, the introduction of new referents complicated things. The distance between successive signals for the same referent tended to be highest just after a new referent had been introduced. That is, introducing a new referent seems to have destabilized existing systems. To investigate this we used a linear mixed-effects model with auto-distance (distance between the current signal and the last signal for the same referent) as a dependent variable; as fixed effects we had turn number since the last new referent was introduced, condition, and overall turn number, as well as their interactions. We included random intercepts for referent, pair, and sender. There was an effect of turn since last referent, β = −5.83 × 10^−4^, *SE* = 1.02 × 10^−4^, *t*(1.18 × 10^4^) = −5.75, *p* < 0.001, and an effect of overall turn number, β = −3.25 × 10^−4^, *SE* = 3.27 × 10^−5^, *t*(1.17 × 10^4^) = −9.94, *p* < 0.001, but no effect of condition. There was, however, an interaction between the three fixed effects, β = −6.87 × 10^−7^, *SE* = 3.18 × 10^−7^, *t*(1.18 × 10^4^) = −2.16, *p* = 0.03. This suggests that, while turn number (and experience) had an effect on the distance between successive signals, the introduction of new referents was having an effect of its own, distinct from how far into the game participants were.

## 4. Discussion

In this paper we have presented *post-hoc* exploratory analysis of experimental data gathered by Roberts and Clark (2020). In the original experiment, designed to investigate the role of non-modality-specific production–perception dynamics in the emergence of phonological structure, participants played a communicative game in which articulation took the form of finger movements on a trackpad, which produced perceptual signals in the form of colors. The basic results of the original experiment were that patterns of dispersion emerged that strikingly resemble patterns observed in vowelspaces in natural languages (Figure 5) and that this seemed to be *primarily* driven by perceptual demands, but that misalignment of perceptual and production demands made establishing a communication system harder, reducing overall success rates.

In the new analysis we investigated participants' initial strategies, convergence with their partners, and the emergence of dispersion patterns. We found that participants seem to have begun the game by selecting colors at random within the most comfortably accessible area for a right-handed person resting the bottom of their palm near the bottom right of the pad, resulting in positively correlated *x* and *y* coordinates for their signals. However, this pattern broke down as they got more used to the game and established their first successful signals, suggesting that participants had by this point begun to expand the range of their fingers on the pad.^6^ However, even at this point colors were not selected with maximal dispersion in mind; rather, dispersion emerged over time, increasing over approximately the first 75 turns before stabilizing—for the remaining 80% of turns—at roughly half of the maximum dispersion possible for two signals (this pattern was especially pronounced in the Outer-edge condition). Variability in dispersion levels also reduced over time, especially in the Outer-edge condition. This can be observed in the decreasing space taken up by each referent's signals over time. In other words, participants became more reliable as they progressed through the game, especially in the Outer-edge condition where such reliability was more easily afforded while still satisfying perceptual demands.

There are at least two different explanations for participants' increasing reliability. One is that the “phoneme” categories became increasingly entrenched over time through experience, as participants got better at hitting the same place through repetition. Another is that participants simply got more used to the relationship between finger position and underlying color space over time. It is likely that both played a role: It would be surprising if participants did not get better at hitting the same target; it would also be surprising if participants did not also become more familiar with the medium over time; and it would be surprising if both did not lead to greater accuracy. It is, however, difficult to tease the two apart in order to assess which might be playing the bigger role. In future work, this could be investigated by looking at participants' behavior in new tasks in which these factors are isolated from each other (such as by making the color space fully apparent throughout).

To some extent (and primarily in the Outer-edge condition), signals also became more extreme over time, that is, closer to the center and corners of the space. In the Outer-edge condition, the corners and center were especially favored, along—secondarily—with the non-corner edges of the pad. The latter were also favored in the Inner-edge condition, with a much smaller (but still apparent preference) for the center and corners. Finally, self-reliability (or auto-distance) was well-correlated with how reliably participants replicated their partners' signals, and both were correlated with success across and within conditions. Furthermore, participants seem to have paid attention to success: they kept closer to what their partner did last if what their partner did last was successful. This is consistent with existing work on reinforcement learning in development (Goldstein et al., 2003; Kapatsinski et al., 2020).

It is important to recognize that, while the analysis presented in this paper is quantitative, there is—as always in such cases—a substantial qualitative component in the *interpretation*. Furthermore, this represents a *post-hoc* exploratory analysis. It was not planned when the original experiment was conducted and should be taken with more caution than a planned analysis would be. It is presented with the goal of stimulating future research rather than testing any particular hypotheses. Nonetheless, we consider that it presents a compelling picture of the emergence of structure through interaction. In particular it is notable that the observed dispersion seems not to have come about as something participants directly planned (at least not from the beginning); nor, on the other hand, was its emergence unrelated to their goals. Rather, it seems to have emerged as a large-scale epiphenomenal property of the system resulting from smaller-scale deliberate choices (cf. Lindblom et al., 1983; Wedel, 2003; Keller, 2005). To put it another way: Participants brought about dispersion without necessarily aiming directly for dispersion *per se*. This is important because it concerns a fundamental question of language evolution, namely, what is the relationship between individual cognition and the distribution of features across the world's languages? The process by which we get from the former to the latter is not simple and direct; it is an indirect and complex cultural-evolutionary process in which languages adapt to the brains and bodies that are using them and the goals that they are used to serve (Kirby et al., 2004). Furthermore, while this process is often cast as primarily about learning—treating, that is, human generations as the primary locus of cultural evolution—our study provides evidence of this process in interaction (cf. Fay et al., 2010; Galantucci et al., 2012; Hasson et al., 2012).

We do not, however, mean to imply that we consider interaction to be the sole means by which phonological organization, or linguistic structure more generally, comes about. We certainly think it is important, but we also think that other factors, such as the particular structure of the articulatory and perceptual systems, are likely to be quite important (Flemming, 2001; Stevens and Keyser, 2010; Carré et al., 2017), as well as learning, particularly repeated learning over generations (Kirby et al., 2014; Verhoef et al., 2014). In particular, it would be quite important in future work to incorporate non-linear quantal topology into the relationship between finger position and the underlying color space (Stevens and Keyser, 2010). Excitingly, incorporating these elements is well within reach of the paradigm. Indeed, we consider this paradigm to be one that can be extended in quite a range of ways for investigating the emergence of phonological (or quasi-phonological) structure in a way that abstracts away from natural language in order to isolate particular mechanisms and constraints involved (cf. Roberts, 2017). And these are by no means restricted to the dynamics investigated by Roberts and Clark (2020). In the present paper, furthermore, we have expanded the range of analytic approaches that can be brought to bear on the data and have, we believe, shed useful further light on where phonological organization might come from.

## Data availability statement

Data and scripts for the study are available at https://osf.io/3c4zb/; further inquiries can be directed to the corresponding author.

## Ethics statement

The studies involving human participants were reviewed and approved by University of Pennsylvania IRB. The patients/participants provided their written informed consent to participate in this study.

## Author contributions

GR performed the analysis and drafted the manuscript in discussion with RC, who provided critical feedback and conceptual suggestions. All authors contributed to the article and approved the submitted version.

## Footnotes

^1^Pillai scores were introduced for this purpose by Hay et al. (2006) and are probably now the preferred approach to measuring whether two vowels have merged. This statistic represents the proportion of one variance that can be predicted by another variance and ranges from 0 to 1, where a higher number indicates a greater difference between distributions (see DasGupta, 2005, for a formal account and Hall-Lew, 2010, for a description of how to calculate it in R).

^2^This is an exception to the general principle of focusing on *xy* coordinates to the exclusion of RGB coordinates, as the RGB space was sufficiently distinct from the *xy* space to make examining dispersion in both worthwhile.

^3^As noted by Roberts and Clark (2020), p. 129f6, this made a success score of 1 strictly impossible as participants did not see all 12 referents at the start of the game; since the success metric was intended as relative rather than absolute, we did not deem it necessary to complicate the measure by taking this into account.

^4^Roberts and Clark (2020) normalized their measures of overall dispersion (Figure 9) by dividing by the maximum possible dispersion given the number of units in the set. We have not done this here. Indeed, it is interesting that participants succeeded in maintaining a rather constant level of dispersion as the demands acting on their communication system increased.

^5^One potential issue with this approach is that, as the game moves on and participants have more referents to communicate, each referent occurs less during a given quintile, so there are fewer signals for every referent. Potentially this could play a part in reducing apparent variability. To investigate this we normalized for each quintile by taking the largest coordinate cloud that had occurred in any quintile and artificially expanded all coordinate clouds to the same size with randomly generated points generated from the mean and standard deviation of the actual cloud. The resulting pattern was almost identical to the pattern with the real data.

^6^It is important to note that this account is based on an intuitive interpretation of our results, rather than a systematic attempt to observe participants' behavior. In future work, this question would be interesting to investigate more precisely.
