# Supplementary material for: The emergence of phonological dispersion through interaction: an exploratory secondary analysis of a communicative game
Source: Front Psychol. 2023 May 24;14:1130837. doi: 10.3389/fpsyg.2023.1130837 (PMC10244737; doi:10.3389/fpsyg.2023.1130837)
Supplement: Supplementary file 1 [file Data_Sheet_1.pdf]

# A Detailed experimental method

## A.1 Participants

Sixty undergraduate students (34 female and 26 male), none of them color-blind, participated in dyads for course credit.

## A.2 Materials

Participants sat in separate cubicles, each with a computer (a mid-2014 Apple iMac with a 21.5″ screen), running custom-designed software written in Python (Python Software Foundation, www.python.org) and Kivy (Virbel, Hansen, & Lobunets, 2011; www.kivy.org), and a wireless multitouch trackpad (a 2009 Apple Magic Trackpad, measuring 13.01 cm by 13.13 cm). Participants could not see each other from their cubicles or hear each other easily.

## A.3 Procedure

Pairs of participants played a cooperative communication game, taking turns to be *Sender* and *Receiver*.[1] Each participant (henceforth *player*) in a dyad sat in a separate cubicle and saw a screen divided vertically into two halves. (For the most part, the screen looked much the same whether the player was Sender or Receiver; Figure 1). In the left half of the screen – the *referent panel* – a set of *referents* were displayed (black animal silhouettes, a subset of those used by Roberts & Galantucci, 2012; Figure 15a).[2] The top right quarter of the screen, the *color panel*, appeared gray by default, but would change color depending on the behavior of the Sender. The same was true of a smaller section immediately below it – the *sent-color panel* – which was also gray by default and took up a quarter of the width of the screen as a whole and a quarter of the height. (See Section A.4 below for a description of how the color panel and the sent-color panel worked.) To the right of the sent-color panel, a timer was displayed on a white background. Below this, taking up half the width of the screen, was a *score panel* displaying the dyad's joint score against a black background.

The referent panel differed slightly for the Sender and the Receiver. First, the referents were not in the same places (i.e., were redistributed at random) from round to round. Second, no referent was ever in the center of the Receiver's referent panel; the Sender, on the other hand, always had one referent in the center, against a red background (Figure 1). This varied from round to round and was selected at random, by the server, from the set of available referents. Third, the Receiver had a green cursor that could be moved around the referent

[1] It was important for our question that both members of each dyad have an equal opportunity to be Sender and Receiver. Had this not been the case, any differences between conditions might be explicable in terms of a failure on the part of the Sender to appreciate the Receiver's needs. This approach also had the advantage of greater ecological validity.

[2] Roberts and Galantucci (2012) used 20 referents in total; we used a 12-referent subset of theirs in order to give participants time to refine their signaling systems. Given the time available, continuing to add referents until there were 20 would have meant that systems would be in a constant state of flux.

panel by using the arrow keys on the computer keyboard (Figure 1b); the Sender had no such movable cursor.

The Sender's task was to convey to the Receiver which referent was highlighted in the center of their referent panel by sending series of colors to the Receiver (see Section A.4 below), and the Receiver's task was to move their cursor to the correct referent and press enter. Both players would then receive feedback: The correct referent would be highlighted in the referent space for the Receiver and the chosen referent would be highlighted for the Sender. This happened whether or not the Receiver chose correctly. If the Receiver did choose correctly, the dyad would score one point; their total point score was displayed throughout the game in the score panel at the bottom of the screen. After players started to do well at signaling the referents, more were added, in groups of four, up to a total of twelve. This would occur if, for all referents in the referent panel, the Receiver had selected them correctly at least 75% of the time over the previous four rounds in which they had occurred (cf. Roberts, Lewandowski, & Galantucci, 2015). Once referents were added, they were never removed and would continue to occur as targets, even if players started to do badly.

A round lasted 20 seconds in total, with feedback lasting an additional 2s. If the Receiver had not chosen a referent by the time the 20 seconds were up, the dyad scored no point for that round. Whatever the outcome of the round, the players would swap roles for the following round. The game lasted for 80min in total, and would finish at the end of the current round when the 80min mark had been passed. At the start of the experiment, players played four practice rounds that differed from the ordinary rounds in three ways: First, they lasted 60 s rather than 20 s; second, the players' score from these rounds did not carry over into the normal rounds; third, players were reminded at the start of each round whether they were Sender or Receiver. Beyond being told to move a finger around the pad and observe the screen, and to hold a finger down for 1s to send a color, players were not instructed how to use the signaling medium, but rather had to explore it on their own.

## A.4 Signaling medium

To convey to the Receiver which referent to select, the Sender could send a series of colors. This could be achieved by moving one finger around on the trackpad, which would produce a color in the color panel on the top right of the Sender's (though not Receiver's) screen, which would change in real time depending on the coordinates of the Sender's finger. If the Sender took their finger off the pad or touched the pad with more than one finger, the color panel would appear gray. If the Sender held their finger in place on the trackpad for 1s or longer, the same color would appear for 2s both on the Receiver's color panel and on the Sender's sent-color panel. (This 2s period was fixed and was not influenced by how long the Sender held their finger down; in other words, duration was not a variable property of the color units.) This was the only means by which the Sender could send information to the

Receiver.[3] A Sender could send as many colors as they liked – including none at all – within the time available (20 s).

The relationship between the Sender's finger position and the color produced was based on an RGB color space, with each color composed of a red, a green, and a blue component, the contribution of each ranging from 0 to 1 (e.g., the vector [1, 0, 0], where the digits indicate the red, green, and blue components respectively, would correspond to a bright red color). The basic value for one of the three components increased from 0 to 1 as the Sender's finger moved from right to left on the pad, while another decreased from 1 to 0 in the same direction; the third color component increased as the finger moved vertically. Which color corresponded to which direction was counterbalanced between dyads, but for any given dyad, the exact center of the pad corresponded to the vector [0.5, 0.5, 0.5]. If vertical position corresponded to the blue component, then placing the finger in the middle of the top edge of the pad would produce an equal mixture of red and green [0.5, 0.5, 0], while the middle of the bottom edge would produce a mixture of red, green, and blue, with blue predominating: [0.5, 0.5, 1]. Players were not in fact exposed precisely to the basic color values described here; instead, the values were modified in a way that varied between two conditions. The details of this are described in Section A.5.

## A.5 Conditions

There were two conditions. In the *Outer-edge* condition the basic color values described above were altered depending on how close the Sender's finger was to the center of the pad (Figure 2). This was done by multiplying the color component values by a modifier that ranged from 0 to 1. The modifier was calculated as $d/d_{oe}$, where $d$ equals the Euclidean distance between the Sender's finger and the center of the space and $d_{oe}$ equals the distance from the center of the space to the outer edge. This meant that colors towards the outer edges of the space were likely to be easier for the Receiver to distinguish. Since the edges of the pad were also easier to find reliably for the Sender, the pressures acting on the Sender and Receiver were therefore relatively aligned in this condition. Figure 15b shows an example of a "word" created in the Outer-edge condition (for a dyad whose color space was as in Fig. **??**).

In the *Inner-edge* condition this was not the case. Here, an imaginary line was drawn 30% of the way in from the edge of the pad. Between the real edge of the pad and this "inner edge", the modifier was calculated as $1 - (d/d_{oe})$. Once the Sender's finger crossed the inner edge, however, the modifier changed to $d/d_{ie}$, where $d_{ie}$ is the distance from the center of the pad to the inner edge. This meant that the colors got brighter as the Sender's

[3] In this respect, our study differs from earlier laboratory-language studies on combinatorial systems, which were mostly concerned with investigating the emergence of atomic units from continuous media. Participants in those studies were thus not provided with preordained means of producing units, with the consequence that identifying how such units might be constituted is itself a challenging task (Roberts & Galantucci, 2012). Because of this, and because we were concerned not with the emergence of such units, but how they become organized, our task forced subjects to select units from a continuous space, thereby simplifying our analysis while still retaining a continuous signal space from which units could be drawn.

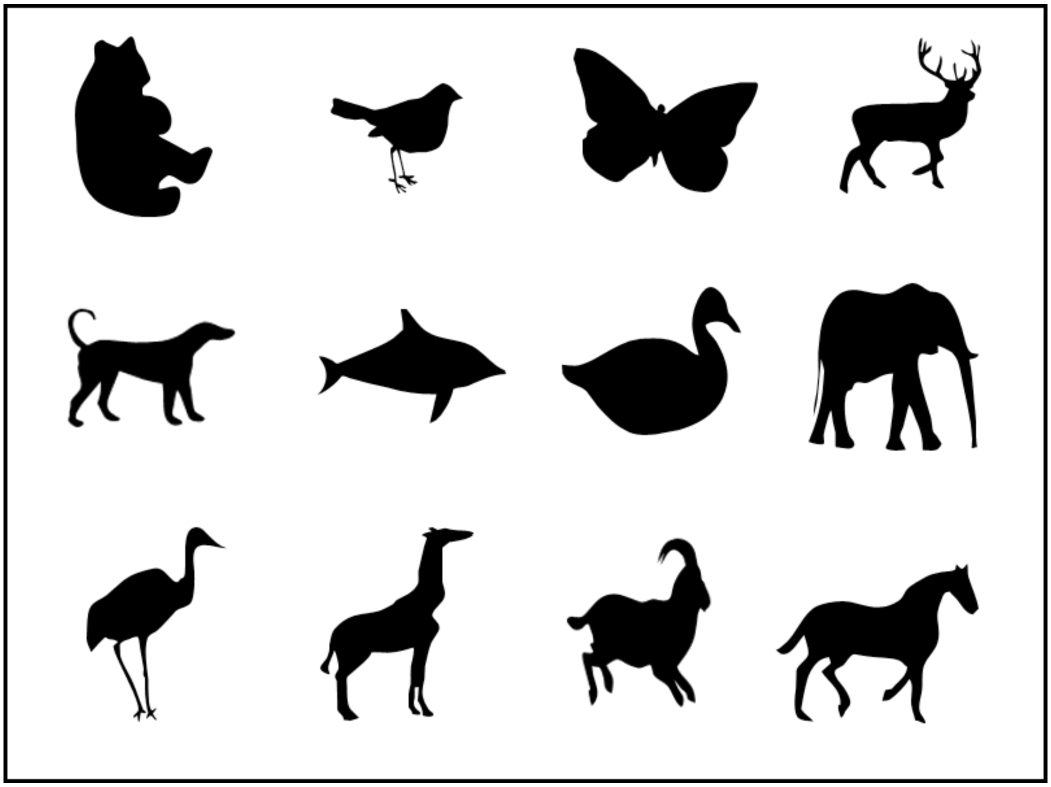

(a) Referents used in the experiment. The top row appeared at the beginning of the game

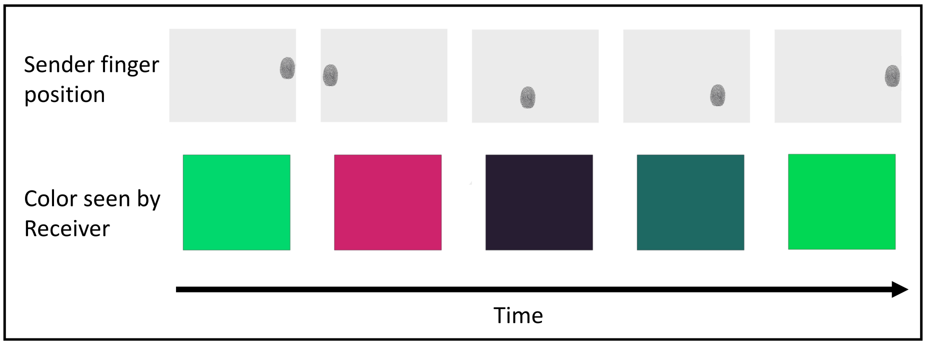


(b) Example five-phoneme word. Fingerprint indicates finger position; color indicates resulting color, consistent with Figure 2 (Outer-edge condition).

Figure 15: Referents and example signal from original experiment.

finger moved away from the center of the pad, but then began abruptly to get darker again. The most convenient parts of the pad for the Sender to select reliably were still along the outer edge of the pad, but the easiest colors to distinguish for the Receiver were closer to the inner edge. The inner edge was in no way marked on the pad or screen; it became apparent to the Sender as they moved their finger around the pad and observed the effect.

# References


Roberts, G., & Galantucci, B. (2012). The emergence of duality of patterning: Insights from the laboratory. *Language and Cognition*, *4*(4), 297–318. doi: 10.1515/langcog-2012-0017

Roberts, G., Lewandowski, J., & Galantucci, B. (2015). How communication changes when we cannot mime the world: Experimental evidence for the effect of iconicity on combinatoriality. *Cognition*, *141*, 52–66. doi: 10.1016/j.cognition.2015.04.001

Virbel, M., Hansen, T., & Lobunets, O. (2011). Kivy – A framework for rapid creation of innovative user interfaces. In *Workshop-Proceedings der Tagung Mensch & Computer 2011. überMEDIEN|ÜBERmorgen.*
